# The Influence of Group Psychology on Network Cluster Behavior: A Moderated Mediation Model

**DOI:** 10.3390/bs16030465

**Published:** 2026-03-20

**Authors:** Jianjun Ni, Zhangbo Xiong, Mingzheng Wu

**Affiliations:** 1 Teacher Work Department, Zhejiang A&F University, Hangzhou 311300, China; 2 School of Education, Shanghai Normal University, Shanghai 200234, China; 1000549999@smail.shnu.edu.cn; 3 Department of Psychology and Behavioral Sciences, Zhejiang University, Hangzhou 310058, China; psywu@zju.edu.cn

**Keywords:** group psychology, opinion leaders, group polarization, network cluster behavior

## Abstract

With the rapid development in new media and social platforms on the internet, some social hotspots or sensitive events can easily ferment and spread in the online space, attracting the attention or concentrated discussion of young students. Network cluster behavior is a collective behavior in which a large number of netizens collectively express and gather opinions around social hot issues of common concern, creating online public opinion. The study explored the influence of group psychology on the process of college students participating in online cluster behavior. A survey was conducted involving 2137 college students from over 10 universities in Zhejiang Province, Jiangsu Province, and other regions. The data were analyzed using correlation analysis and moderated mediation model testing. This study found that group psychological factors, such as emotional infection, depersonalization, the spiral of silence, relative deprivation, group polarization, and action mobilization, positively predicted network cluster behavior. The action mobilization of opinion leaders mediated the relationship between emotional infection and network cluster behavior. Group polarization mediated the relationship between the spiral of silence and network cluster behavior. Additionally, group efficacy moderated the latter part of the mediation process between group polarization and network cluster behavior.

## 1. Introduction

Currently, complex social contradictions often underpin various social hot spots or sensitive events. When these latent contradictions and conflicts manifest as explicit social events, they can readily precipitate actual network cluster behavior. The advent of new media has transformed people’s living habits and methods of information exchange, while also altering and expanding the scope and media range of network cluster behavior, potentially resulting in large-scale network cluster behavior (Ellison et al., 2007). College students, as a significant demographic within the network society, are increasingly aware of their role in democratic participation in public affairs due to the rapid development of new media platforms and information technology (Ferreira & Menezes, 2021). By engaging with national political and social developments, these students discuss socially sensitive events through online group interactions, articulate reasonable suggestions and interests, and thereby contribute to the formation of network public opinion and the occurrence of network cluster behavior (Literat & Kligler-Vilenchik, 2019). Robert Park, an American scholar, first proposed the concept of cluster behavior in 1921 and, from a sociological perspective, believed that cluster behavior was an individual behavior under the influence of collective impulse (Park & Burgess, 1921, p. 865). Network cluster behavior refers to the gathering behavior of a large number of netizens who participate in interactive group discussions, express and gather opinions on a large scale, shape online public opinion, and influence real-life events related to social phenomena or hot issues of common concern, often for specific purposes or interests, within the online space (Loader et al., 2014).

In the governance of cyberspace and the construction of cyberspace culture, it is necessary to cultivate a positive and healthy cyberspace culture so as to create a clean and positive cyberspace for the majority of Internet users, especially teenagers. Network clustering behavior has both positive and negative effects. With the rapid development and application of new media platforms, sensitive issues and conflicts in real society can easily trigger negative online public opinion. Compared to others, some negative views, attitudes, and emotions are more likely to ferment and spread on social media platforms, affecting social stability and requiring the attention of the government and universities (Bu, 2020). College students represent a primary participant group in network cluster behavior. Negative online public opinion events can adversely impact their healthy development and disrupt both the order of cyberspace and the harmony and stability of campuses. Methods for enhancing the governance capabilities and modernization of governance systems in cyberspace are also a critical issue that the current government and universities need to address (Bu, 2020).

## 2. Literature Review and Research Hypotheses

### 2.1. Theoretical Basis for the Formation of Network Cluster Behavior

With the rapid development of new online media and social platforms, the “Internet plus” model has changed the thinking habits and lifestyle of college students (Zhao, 2021). In particular, once some issues involving the interests of students (such as emotions, examinations, employment, etc.) are published and disseminated online, they are likely to attract college students’ attention and resonance. Through WeChat, Tiktok and other channels, they can make comments and express appeals, quickly ferment, spread and trigger online public opinion, and even trigger network cluster behavior in colleges and universities, affecting campus security and stability. The psychology of online groups has characteristics such as anonymity, infectiousness, suggestion, and extremism. It is a common psychological state among members of online groups, which can easily lead to irrational group behavior (Ran & Zeng, 2023).

American sociologist Neil Smelser proposed that the occurrence of cluster behavior requires six conditions: environmental conditions, structural tension, the generation of universal emotions and the formation of shared beliefs, triggering factors, mobilization of participants’ actions, and social control (Smelser, 1963, p. 8). French psychologist Gustave Le Bon conducted in-depth research on the phenomenon of group psychology in his book “*The Crowd*” and believed that when individuals gather together, their psychological state changes, personality gradually disappears, and group thinking and concepts converge in the same direction, forming collective psychology (Le Bon, 1895).

This study is based on the Social Identity Theory (SIT) proposed by social psychologist Henri Tajfel as the supporting theory for this research (Tajfel & Turner, 1979). Social identity theory is one of the most representative theories for studying group relationships, aiming to explain the psychological processes and behavioral patterns of individuals in a group context. The social identity theory holds that the emergence and development of cluster behavior are related to group identity. When an individual’s social identity is threatened, based on the motivation to maintain self-esteem, they will use various methods to regain social recognition. At this time, vulnerable groups may generally resist threats and oppose social injustice through group behavior (van Zomeren et al., 2008). When a large number of netizens gather and express their opinions on social hot issues of common concern on social media platforms, creating online public opinion, it will trigger online clustering behavior.

This article combines Gustave Le Bon’s relevant characteristics of group psychology, focusing on analyzing the reasons and social psychological mechanisms of network cluster behavior (collective action based on common social identity) from the perspectives of group psychology, such as the spiral of silence (group comparison and pressure), relative deprivation (negative social comparison), depersonalization (personal identity turning to group identity), emotional contagion (shared emotional formation), action mobilization (framing identity of opinion leaders), group polarization (identity extremism and position strengthening), and group efficacy (collective action belief) (Drury & Reicher, 2005). This study integrates relevant variables into a coherent logical chain from “psychological identification” to “overt behavior” through social identity theory, providing a profound theoretical explanation for network cluster behavior.

At present, the research on network cluster behavior in academia is showing a multidisciplinary trend and has formed a certain theoretical basis and research results, but there are also some shortcomings. From the analysis of literature, it can be seen that most existing studies have borrowed traditional communication theories to analyze network cluster behavior and its online public opinion, and most of the research is limited to studying from the perspectives of sociology, political science, management, news communication, etc., while empirical research from social psychology is relatively scarce (Nie & Lü, 2021). There are a few existing studies that use group psychological factors such as the spiral of silence, relative deprivation, depersonalization, emotional contagion, group polarization, and group efficacy as research variables to analyze network cluster behavior. In particular, research on the group psychological characteristics, social psychological mechanisms, and evolutionary mechanisms in the formation process of network cluster behavior is not yet in-depth enough (T. T. Wang et al., 2025).

### 2.2. The Influence of Group Psychology on Network Cluster Behavior

#### 2.2.1. The Relationship Between the Spiral of Silence and Network Cluster Behavior

The spiral of silence was first proposed by German scholar Elisabeth Noelle-Neumann. The spiral of silence refers to the tendency of participants to express their ideas more boldly and actively when they see that their views and attitudes align with the opinions of the majority of the group or are praised during discussions on a controversial topic related to hot and sensitive events (Noelle-Neumann, 1984); But when he finds that his views and attitudes are inconsistent, even opposite or conflicting with the opinions of the majority of people in the group, he will show caution and even experience group pressure and psychological anxiety. In order to avoid being isolated or verbally attacked by the group, he will change his original opinions and attitudes or choose to remain silent (Zou et al., 2025). This will lead to a development process where one party’s strong viewpoint continues to strengthen, while the other party’s weak opinion sinks and weakens, forming a spiral downward trend (Noelle-Neumann, 1984). In the process of group discussions on network cluster behavior, individuals often observe the overall views and attitudes (opinion climate) of other members of the surrounding network group first due to fear of being isolated and feeling group pressure, make their own judgments, identify and evaluate them, and then express their opinions to ensure their psychological safety in the group (Scheufele, 2008).

#### 2.2.2. The Relationship Between Relative Deprivation and Network Cluster Behavior

Relative deprivation can lead to cognitive biases and negative emotional experiences in individuals (De La Sablonnière & Tougas, 2008), manifested in subjective psychological experiences of feeling “deprived” compared to their reference group. When individuals feel “deprived”, they may exhibit negative emotions and psychological experiences such as inferiority, repression, resentment, dissatisfaction, anger, and even extreme speech or violent behavior (H. J. Smith et al., 2012). College students with a strong sense of relative deprivation tend to frequently compare their studies and lives with those of their classmates around them, often leading to negative emotions such as inferiority, loss, dissatisfaction, resentment, and even aggressive behavior (J. Chen & Xiong, 2023). In the online social space, college students with a relatively strong sense of deprivation are more likely to resonate, thereby promoting the occurrence of online cluster behavior (Wan et al., 2023).

#### 2.2.3. Relationship Between Depersonalization and Network Cluster Behavior

De individualization was first proposed by French social psychologist Gustave Le Bon, which reduces individual psychological anxiety and self perception in reality, manifested in the loss of identity characteristics such as gender, age, occupation, and social status in group activities (Y. X. Chen et al., 2019), and the phenomenon of individuals being submerged in the group and losing their sense of individuality (Valkenburg & Peter, 2011). Due to the relative anonymity of virtual spaces on the internet, individuals’ sense of identity and self perception in reality have decreased (Baumeister & Vohs, 2007). Compared to real-life situations, individuals’ psychological anxiety, group psychological pressure, and sense of social responsibility in online social virtual spaces will decrease, making it easier to trigger online cluster behavior (Lambert et al., 2000).

#### 2.2.4. The Relationship Between Emotional Infection and Network Cluster Behavior

The concept of emotional contagion was first proposed by American psychologist William McDougall in 1923 as a phenomenon of emotional aggregation. Emotional contagion can enhance an individual’s sense of group belonging and psychological suggestion (van Kleef & Côté, 2022), specifically manifested in similar situations where individuals with similar value orientations, identity status, life experiences, professional values, and personality traits, under non coercive or unconscious conditions, resonate emotionally, identify psychologically, and communicate emotionally with certain specific events (Le Bon, 1895). The emotional cognition of netizens is an important emotional factor in the formation process of online cluster behavior, driving the development of public opinion (Zhao et al., 2024). College students with high levels of emotional contagion are more likely to actively accept and imitate the ideas, attitudes, and behaviors of other members in the online community when hinted at by others (Coviello et al., 2014). Individual emotions will spread with the dissemination of information, promoting the cohesion and resonance of group emotions, and driving the occurrence of network cluster behavior.

#### 2.2.5. Mediating Role of Group Polarization

Group polarization was proposed by American scholar James A.F. Stoner in 1961 when studying the impact of group discussions on individual and group decision-making (Sunstein, 2009). Group polarization refers to an individual’s psychological perception of an extreme tendency towards the viewpoints, attitudes, and decision-making judgments of group members. After exchanging opinions and emotional contagion in network cluster behavior, group views become more biased and inciting. Group polarization can lead to the phenomenon of responsibility dispersion and risk transfer (Myers & Bishop, 1970). In the process of college students participating in group discussions on hot and sensitive topics or negative public opinion events on the internet, due to group pressure and fear of being isolated by the group, they actively observe and judge the opinions and attitudes of other people around them. When they see that their own views are consistent with the opinions of the majority in the group, they will take the initiative to speak boldly and express their own ideas; When one finds that their viewpoint differs from that of the majority in the group, they may speak cautiously or choose to remain silent (E. M. Smith et al., 2021). With the rapid rotation of the ‘spiral of silence’, the dominant views of the majority are becoming stronger, while the weaker views of the minority are becoming weaker, ultimately leading to a ‘polarization’ phenomenon in group discussions (Lu et al., 2023). Under the influence of group polarization, group members tend to make more risky decisions, which affects the formation of network cluster behavior (Amelkin et al., 2017).

#### 2.2.6. The Regulatory Effect of Group Efficacy

Group efficacy refers to the individual members’ judgment of the collective ability of the group they belong to or their evaluation of the ability to complete a certain task, specifically manifested in their psychological perception of the group’s ability to unite and solve practical problems. When group members evaluate that the team’s ability may change in unfavorable situations, they are more inclined to participate in network cluster behavior (Bandura, 2000). Individuals with a strong sense of group efficacy have a stronger willingness to create social public opinion through participating in network cluster behavior to achieve group-related interests and demands (Kelloway et al., 2007). Some sensitive topics or negative public opinion events on the internet, after group discussions, are influenced by the “spiral of silence”, leading to a polarization of opinions and viewpoints within the group (Meyer et al., 2022), ultimately resulting in more risky and bold judgments and decisions. At the same time, influenced by the “superposition effect” of group efficacy, there is a greater tendency to participate in network solidarity, concentrate on expressing interests and demands, leading to the occurrence of network cluster behavior (Badea et al., 2021).

#### 2.2.7. The Mediating Role of Opinion Leaders

Opinion leaders were proposed by American communication scholar Paul F. Lazarsfeld in the 1940s, who believed that in the process of mass communication, information flows to the audience through the intermediate link of opinion leaders (Jain, 2022). Opinion leaders are a key factor in the behavior of online clusters, playing a mobilizing role in the development of public opinion (J. Wang et al., 2018). Action mobilization refers to the process in which online opinion leaders and others purposefully guide or organize the mobilization of netizens to participate in online cluster behavior. Opinion leaders are the communication hub of online cluster behavior, playing a key role in the development of online public opinion and the emotional evolution of netizens (Turcotte et al., 2015). Young students who are emotionally infected are more inclined to accept or imitate the attitudes and behaviors of other members in online groups. Opinion leaders generally have high discourse power and influence in online communities (Tobon & García-Madariaga, 2021), and are also a key factor influencing online public opinion and online cluster behavior (J. Wang et al., 2018).

### 2.3. Research Hypothesis

Based on the previous literature review and the theoretical foundation of network cluster behavior formation, as well as the influence of group psychology and network cluster behavior, such as the spiral of silence, relative deprivation, depersonalization, emotional contagion, group polarization, opinion leaders, and group efficacy, the following research hypotheses are proposed:

In the process of individual participation in network cluster discussions, as a “spiral of silence” forms, the party with weaker opinions, due to group pressure, fears being isolated by the group and becomes silent, leading to convergent behavior. As a result of group discussions, the dominant opinion of one party becomes dominant (Sohn & Geidner, 2016). At this point, there is a relatively consistent opinion within the group, and even a phenomenon of “polarization” may occur. Group members are prone to making bold decisions or taking risks, which can trigger network clustering behavior (Wallach et al., 1962). Based on this, hypothesis H1 is proposed: the spiral of silence has a positive impact on the occurrence of network clustering behavior.

In the process of participating in online group discussions on sensitive topics or negative events, especially when individuals or groups with similar backgrounds and experiences are infringed upon or ‘deprived’ of their interests, they are more likely to resonate, become agitated and angry, participate in online solidarity and collective rights protection, and even participate in real-life collective actions (Foster & Matheson, 1995). Based on this, hypothesis H2 is proposed: relative deprivation has a positive impact on the occurrence of network clustering behavior.

Due to the relative anonymity of the virtual space of online social interaction, individual identity characteristics are discussed, and the constraints of online behavior are reduced. College students are more likely to express excited opinions or critical viewpoints when participating in online group discussions. At the same time, due to factors such as lack of responsibility and dispersed responsibilities in online groups, college students are also more likely to participate in online group behavior (van Kleef & Côté, 2022). Based on this, hypothesis H3 is proposed: Depersonalization has a positive impact on the occurrence of network clustering behavior.

For the hot sensitive topics or negative events discussed by groups on new online media such as WeChat and Tiktok, especially when seeing the damage to individual interests similar to their own life experience or value orientation, they are more likely to feel empathy, emotional excitement and generate psychological resonance (N. Wang, 2021). Emotional contagion is a “catalyst” (Reimert et al., 2013), which makes them more inclined to actively express dissatisfaction and interest demands on the internet, and participate in network cluster behavior (Wróbel & Imbir, 2019). Based on this, hypothesis H4 is proposed: Emotional contagion has a positive impact on the occurrence of network clustering behavior.

Under the influence of the ‘spiral of silence’, opinions in group discussions may become polarized. Under the influence of group polarization, college students are more inclined to make risky and bold judgments and decisions after group discussions, leading to the occurrence of online cluster behavior and even participation in real-life collective actions (Wei, 2021). Based on this, hypothesis H5 is proposed: group polarization is a mediating variable between the spiral of silence and network cluster behavior. Hypothesis 5 (H5) consists of two components: H5(a): the spiral of silence positively predicts group polarization; H5(b): group polarization positively predicts network cluster behavior.

Group efficacy is closely related to collective action and will change with the relationships between group members and external influences (Yuan & Liu, 2022). Under the spiral of silence, opinions and attitudes within a group may become polarized, leading to more risky collective decisions (the result of polarized opinions and attitudes) and even triggering network cluster behavior. In this process, the level of group efficacy may affect the “strength” of the relationship between group viewpoint and attitude polarization (polarization tendency) and the implementation of collective behavior (the result of group decision-making) (Yin & Fei, 2024). College students with high group efficacy tend to believe or be more inclined to believe that, through the joint participation and collective action of online groups (Drury & Reicher, 2000), events can be effectively promoted and resolved to achieve the interests and demands of their online groups (Zhang, 2013). Based on this, hypothesis H6 is proposed: group efficacy enhances the positive relationship between group polarization and network cluster behavior, that is, the second half of the mediating path from the spiral of silence to group polarization and network cluster behavior is regulated by group efficacy.

Opinion leaders play an important driving role in the formation and development of online cluster behavior and the diffusion of public opinion (H. M. Wang et al., 2024). Under the promotion and organizational mobilization of online opinion leaders, emotionally infected college students are more actively participating in online cluster behavior, joining forces to express their dissatisfaction and seek social support, in order to achieve group interests and demands (Tobon & García-Madariaga, 2021). The call and mobilization of opinion leaders may accelerate the practical transformation from “common emotions” to “collective behavior”. Based on this, hypothesis H7 is proposed: the action mobilization of opinion leaders is a mediating variable between emotional contagion and network cluster behavior. Hypothesis 7 (H7) consists of two components: H7(a): emotional infection positively predicts action mobilization; H7(b): action mobilization positively predicts network cluster behavior.

**Figure 1:**
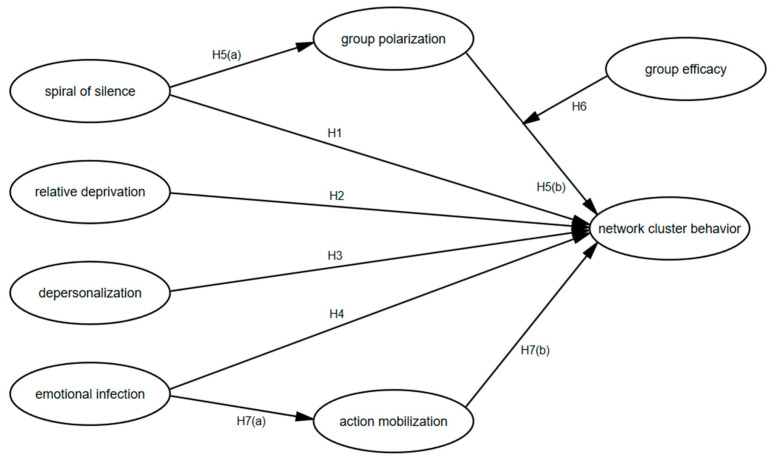
The hypothesized model.

Overall, based on the previous theoretical analysis and inference, this study hypothesizes that factors such as the spiral of silence, relative deprivation, depersonalization, and emotional contagion in group psychology have a positive impact on the occurrence of network cluster behavior. Group polarization mediates the relation between the spiral of silence and network cluster behavior. Group efficacy moderates the relationship between group polarization and network cluster behavior. The mobilization actions of opinion leaders are a mediating variable between emotional contagion and network cluster behavior. Based on this, we construct a moderated mediation model, as shown in Figure 1. This study investigates the psychological characteristics and psychosocial mechanisms underlying college students’ participation in network cluster behavior groups.

## 3. Research Methods and Design

### 3.1. Participants and Data Collection

The sample for this study comprises college students across more than 10 institutions, both within and outside the province. This included 985 project universities, general undergraduate institutions, and higher vocational colleges, spanning various disciplinary backgrounds such as teacher training, science and technology, finance, medicine, agriculture and forestry, and media and art. The data were collected through online questionnaires. A total of 2391 questionnaires were returned; 254 were excluded due to invalid responses, leaving 2137 valid responses, resulting in an effective response rate of 89.4%. The survey was conducted from 1 May 2023 to 7 May 2023.

The characteristics and distribution of the questionnaire sample are as follows: 760 male students (35.6%) and 1377 female students (64.4%); 1657 members of the Communist Youth League (77.5%), 286 members of the general populace (13.4%), and 194 members of the Communist Party of China (9.1%); 1020 students majoring in science and engineering (47.7%), 441 in the literature and history (20.6%), 174 in medicine (8.1%), and 327 in other fields (15.3%); 633 students from large and medium-sized cities (29.6%) and 1504 from rural areas or small towns (70.4%). This distribution indicates a broad and representative sample.

### 3.2. Measurements

#### 3.2.1. Questionnaire Design and Development

The questionnaire comprised two sections: basic information and question items. The first section gathered basic demographic data, including gender, political affiliation, ethnicity, academic year, major, educational background, family origin, and whether the respondent held a student leadership position. The second part is the content of the questions, mainly including network cluster behavior (example question: I will forward or comment on social hot topics discussed by netizens to express opposition or support), the spiral of silence (example question: After a network hot topic event triggers a discussion, I will try my best to maintain consistency with the opinions and views of people around me to avoid being excluded or isolated), relative deprivation (example question: I always feel that others have something that should belong to me), depersonalization (example question: I think I can better express my personality and ideas in virtual network communities than in real life), emotional contagion (example question: When discussing negative network group events, seeing someone angry and upset about it, I may also be provoked) Group polarization (example topic: When discussing controversial topics in online groups, I may appear excited and even have some extremist ideas due to the participation and support of group members), mobilization of opinion leaders (example topic: I enjoy analyzing, commenting, and judging on sensitive events at home and abroad online, fully expressing my own views and unique insights), group efficacy (example topic: For some online hot topics, I believe that through the participation and support of our netizens, we can effectively safeguard the rights and interests of relevant groups), and other dimensions.

It should be noted that in this study, group polarization is judged and evaluated by observing individual psychological perception states. Group efficacy is the analysis and evaluation of group efficacy and collective ability through observing individual psychological perception states. The spiral of silence is based on the communication theory proposed by Elisabeth Noelle Neumann, in which individuals perceive the opinions and attitudes of surrounding members during group discussions and interactions around a common hot topic, and make choices about their own opinions and thoughts after judgment and evaluation (actively speaking out or remaining silent). By observing the individual’s psychological attitude perception state, the overall opinion and attitude (opinion climate) of the group is analyzed and evaluated.

The questionnaire adopts a Likert 5-point scale format, and the more the content of the questions matches, the higher the score. When answering questions, evaluate and judge the questions expressed in the items based on one’s actual situation, with different numbers indicating the degree of conformity between these questions and one’s own reality. Among them, 1 = “completely inconsistent” and 5 = “completely consistent”. To ensure content validity during the scale design process, the following methods were employed: (1) semantic correction and (2) environmental applicability correction. Experts in psychology, sociology, and ideological and political education, both within and outside the university, in addition to representatives from relevant faculty and students, were consulted to review and refine the content of the question items. A preliminary version of the questionnaire titled “Psychosocial Motivation of University Participation in Online Cluster Behavior” (comprising 20 questions) was used in a small-scale pre-survey. Based on the preliminary data, the questionnaire was revised and improved.

#### 3.2.2. Exploratory Factor Analysis

In this study, the questionnaire was revised using SPSS 26.0 and Amos 23.0. Initially, a suitability test was performed, revealing a KMO test value of 0.912 and Bartlett’s test of sphericity chi-square coefficient that reached significance, indicating that the data were appropriate for factor analysis. Subsequently, questions with standardized factor loading coefficients below 0.5 were removed, and the attribution of questions across different dimensions was revised and adjusted to finalize the questionnaire. Thirdly, the final scale retained 20 items, with all item factor loadings above 0.7, and the communalities of the common factors are essentially above 0.75.

#### 3.2.3. Confirmatory Factor Analysis

**Figure 2:**
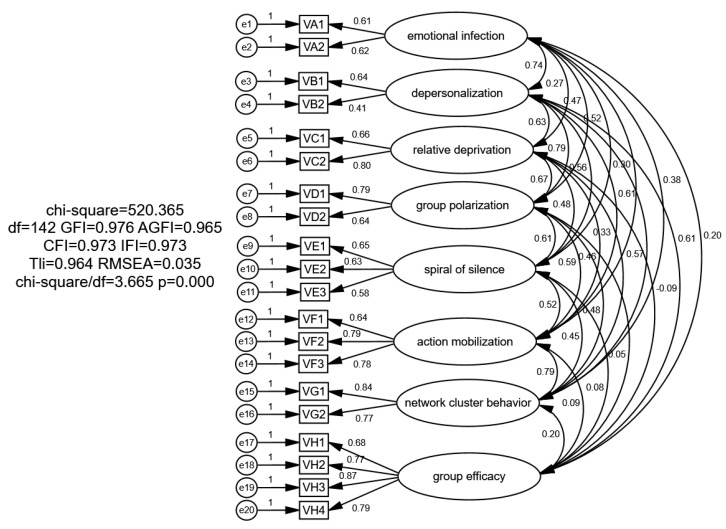
Confirmatory factor analysis (standardized parameter estimates).

A confirmatory factor analysis was conducted on the sample data from the second survey, with the model fit indicators presented in Table 1. The confirmatory factor analysis model is depicted in Figure 2. The χ^2^/df ratio was 3.665, and the values for GFI, AGFI, IFI, CFI, and NFI were all above 0.9. The RMSEA value was 0.035, and the RMR value was 0.025, both of which are below 0.05, indicating a good model fit. Additionally, the factor loadings are generally above 0.7, indicating that the scale possesses good reliability and validity.

#### 3.2.4. Reliability Analysis

This study utilized a self-administered questionnaire, and the reliability analysis showed that the Cronbach’s alpha coefficient for each dimension exceeded 0.7, with the overall reliability of the scale reaching 0.843. These results indicate that the questionnaire has strong overall reliability and that the items possess good internal consistency.

#### 3.2.5. Common Method Bias

Due to the use of questionnaire survey methodology in this study, potential common method bias (CMB) issues were addressed through unrotated principal component factor analysis. The results revealed five factors with eigenvalues greater than 1, among which the first factor accounted for 26.063% of variance—a value below the 40% critical threshold (Wen & Ye, 2014). This indicates no significant CMB effect in the current research.

#### 3.2.6. Ethics Statement

The full name of the ethics committee is as follows: Medical Ethics Committee of the Department of Psychology and Behavioral Sciences, Zhejiang University. Informed written consent was obtained for this study. During the data collection process, consent forms were provided together with the survey questionnaire, detailing the research objectives, content, and target population, and ensuring that participants were aware that they had the right to withdraw from the study at any time. This survey was completely anonymous, did not require any personal information (such as name), and was voluntary.

## 4. Results

### 4.1. Descriptive Statistics and Correlation Analysis

The descriptive statistics and correlation matrix for each study variable are presented in Table 2. The analysis indicates that the spiral of silence, depersonalization, emotional infection, relative deprivation, group polarization, and action mobilization were all significantly and positively associated with network cluster behavior.

### 4.2. Testing the Mediating Effect of Opinion Leaders

Opinion leaders are key drivers of online cluster behavior and play a mobilizing role in the development of public opinion (mediating effect). The mediating effect is analyzed according to the process suggested by Zhonglin Wen and Baojuan Ye et al.: (1) regression of the dependent variable on the independent variable, coefficient c. If c is significant, it is treated as a mediating effect; otherwise, it is considered inconsistent mediation (Wen et al., 2012). Regardless of whether c is significant or not, a subsequent test is performed: (2) the regression of the mediating variable on the independent variable, coefficient a; and the regression of the dependent variable on the independent variable, coefficient c’, and the mediating variable, coefficient b. If both a and b are significant, the indirect effect holds. If c’ is significant, the direct effect holds; otherwise, it does not hold, and only the mediating effect holds. (3) If at least one of a and b is insignificant, the bootstrap test is used, and if it is still insignificant, the analysis is stopped. (4) If both indirect and direct effects hold, the signs of a*b and c’ are compared. If the sign is the same, a partial mediating effect is present; if the sign is different, an inconsistent mediating effect is present.

The results of the mediation effect test are presented in Table 3. There was a significant positive effect of emotional infection on network clustering behavior in model 1 (b = 0.100, SE = 0.022, *p* = 0.000); a significant positive effect of emotional infection on action mobilization in model 2 (b = 0.301, SE = 0.031, *p* = 0.000); and a significant positive effect of action mobilization on network clustering behavior in model 3 (b = 0.392, SE = 0.014, *p* = 0.000), indicating that the mediating effect holds, and hypothesis 7 is supported. The direct effect of emotional infection on network cluster behavior was b = 0.094 (SE = 0.019, *p* = 0.000), while the mediation effect was 0.118, 95% CI [0.097, 0.142]. The consistent direction of both effects indicates that action mobilization plays a partial mediating role between emotional infection and network cluster behavior.

### 4.3. Effect Test with Moderation

First, group polarization and group efficacy are centralized, and then, they are multiplied together to form a product term. Second, the mediated model with moderation was tested, i.e., the mediating effect of group polarization and the moderating effect of group efficacy were analyzed (Walter & Bruch, 2008). We refer the reader to Table 4 for the results of the analysis. In model 1, the spiral of silence significantly and positively affects network cluster behavior (b = 0.169, SE = 0.018, *p* = 0.000), and hypothesis 1 is supported. Relative deprivation significantly and positively affects network cluster behavior (b = 0.098, SE = 0.023, *p* = 0.000), and hypothesis 2 is supported. Depersonalization significantly and positively affects network cluster behavior (b = 0.216, SE = 0.022, *p* = 0.000), and hypothesis 3 is supported. Emotional infection significantly and positively influenced network cluster behavior (b = 0.082, SE = 0.022, *p* = 0.000), and hypothesis 4 is supported. The spiral of silence in model 2 significantly and positively influenced group polarization (b = 0.325, SE = 0.016, *p* = 0.000); group polarization in model 3 significantly and positively influenced network clustering behavior (b = 0.156, SE = 0.025, *p* = 0.000), indicating that group polarization played a mediating effect between the spiral of silence and network clustering behavior, and hypothesis 5 is supported. The direct effect of the spiral of silence on network clustering behavior was 0.142 (SE = 0.018, *p* < 0.001), and the mediating effect was 0.051, with a confidence interval of [0.034, 0.067]. The directions of both effects are consistent, suggesting that group polarization partially mediates the relationship between the spiral of silence and network clustering behavior.

In model 4, the interaction term of group efficacy and group polarization significantly and negatively affected network clustering behavior (b = −0.019, SE = −0.007, *p* = 0.007), and hypothesis 6 is supported. Group efficacy was the moderating variable between group polarization and network cluster behavior, i.e., the second half of the pathway—the spiral of silence → group polarization → network cluster behavior—was moderated by group efficacy.

**Figure 3:**
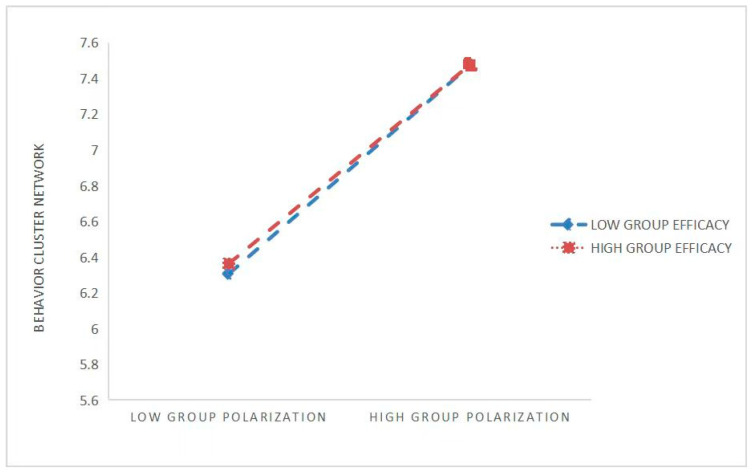
The moderating effect of group effectiveness on the relationship between group polarization and network cluster behavior.

The relationship between group polarization and network clustering under different group efficacy was further analyzed via simple slope analyses according to the mean plus or minus one standard deviation (M ± SD) difference. The mean (M) is the medium-efficacy group, the mean minus one standard deviation (M − SD) is the low-efficacy group, and the mean plus one standard deviation (M + SD) is the high-efficacy group. As observed in Table 5 and Figure 3, when the level of group efficacy is low (−2.6002), the impact of group polarization on network clustering behavior (b = 0.4371, SE = 0.028, *p* = 0.000) is higher than when the level of group efficacy is high (2.6002), where the effect of group polarization on network clustering (b = 0.3448, SE = 0.027, *p* = 0.000) is comparatively lower.

## 5. Discussion

### 5.1. The Relationship Between Group Psychology and Network Cluster Behavior

Emotional infection, depersonalization, relative deprivation, group polarization, spiral of silence, and action mobilization were all found to be significantly and positively associated with network cluster behavior. Meanwhile, research has found that emotional contagion, depersonalization, relative deprivation, group polarization, spiral of silence, and action mobilization can all positively predict network cluster behavior, which is consistent with previous studies (Drury & Reicher, 2005; Y. X. Chen et al., 2019; van Kleef & Côté, 2022; N. Wang, 2021). Among them, the correlation coefficient between the action mobilization of opinion leaders and network cluster behavior is the highest. This indicates that the action mobilization role of opinion leaders is a key factor in triggering and promoting online cluster behavior, playing a crucial role in the formation and development of online public opinion and the emotional evolution of college students (Nian & Zhang, 2025). Similarly, research has found a high correlation between group polarization and the spiral of silence, relative deprivation, action mobilization, and depersonalization. According to the Social Identity Theory (SIT), group polarization refers to the radicalization of identity and the strengthening of positions. This indicates that group polarization is a very important and complex variable in the formation and development of network cluster behavior. In the virtual space of the network, negative psychological experiences such as psychological anxiety and reduced self perception due to depersonalization, depression, dissatisfaction, anger caused by “deprivation”, and group pressure felt during individual interaction and discussion may lead to extreme tendencies in group members’ viewpoints, attitudes, emotions, and group decision-making. The action mobilization and organizational call of opinion leaders during group interactive discussions can also exacerbate the polarization phenomenon within the group (Lan et al., 2019). The interplay among group psychological factors—such as emotional infection, depersonalization, sense of relative deprivation, group polarization, spiral of silence, action mobilization, and group efficacy—plays a crucial role in the participation of college students in online discussions of hot and sensitive topics or negative public opinion events. These factors interact, correlate, and influence each other, collectively driving the formation and development of online cluster behavior. These findings support further exploration of the relationships among these variables, including the analysis and testing of the mediating effects of opinion leaders and group polarization, as well as the moderating effects of group efficacy.

### 5.2. Testing the Mediating Role of Opinion Leaders

This study found that the action mobilization of opinion leaders plays a mediating role in the relationship between emotional infection and network cluster behavior. This indicates that, when college students participate in discussions about trending and sensitive topics or negative public opinion events online, they are prone to emotional infection when they encounter similar experiences or scenarios. This results in emotional resonance, psychological identification, and emotional transfer regarding specific online opinion events. In particular, some students with relatively difficult economic conditions are more likely to have a sense of relative deprivation than other students. When they see negative online events where the interests of disadvantaged groups are harmed, they may feel indignant or even angry (Walter & Bruch, 2008). According to the Social Identity Theory (SIT), when individuals gather based on a common social identity, emotional contagion is highly likely to occur. Shared emotions are the “glue” of group cohesion and the key “fuel” that drives collective action. Emotional infection enhances individuals’ sense of belonging to a group and psychological suggestibility, rendering them more susceptible to adopting and imitating the attitudes and behaviors of other members of the online group. Emotional infection is also affected by different social and cultural differences. Under the influence of traditional Chinese culture, students in the north are relatively resolute, independent, and assertive, while students in the south are more perceptual and easygoing. These cultural differences and personality characteristics will also affect the value judgment of emergencies. When college students perceive that their group’s interests are being infringed upon, they are more likely to proactively post their grievances and interests online to seek support. As these negative emotions accumulate, they can easily result in large-scale network cluster behavior (Tobon & García-Madariaga, 2021).

In the formation and development of online public opinion, opinion leaders, including online vloggers, scholars, and experts, play a significant role. Their expertise, active engagement, and unique perspectives can influence the opinions of other college students. Social Identity Theory (SIT) holds that action mobilization is the framing identification of opinion leaders, which transforms dispersed and vague dissatisfaction into clear and specific collective action goals by setting discussion topics and discourse frameworks. Opinion leaders strategically guide or organize college students into network cluster behavior by mobilizing them to express their grievances and interests online, encouraging collective solidarity, and steering public opinion toward specific objectives. In response to negative online events, opinion leaders monitor the situation, actively post their views and analyses, and stimulate discussions among students. They may even mobilize a larger group of college students to participate in collective actions, thereby fostering online public opinion to ensure fair treatment and the effective resolution of issues (E. R. Smith, 2007).

Regarding social conflicts or sensitive issues, larger groups of Internet users will actively engage in group discussions and express dissatisfaction in achieving specific goals or satisfying interest demands (Bi & Huang, 2020). When the emotional intensity among young student groups reaches a certain level, opinion leaders, through analyzing and commenting on events and expressing their distinctive opinions, play a crucial mediating role in action mobilization. They are more likely to organize and mobilize students to express and consolidate their opinions on a large scale in the online public sphere, thereby influencing or advancing the development of online public opinion in the desired direction and potentially organizing and participating in real-world clustering behaviors (Wu, 2021).

### 5.3. The Mediating Role of Group Polarization and the Moderating Role of Group Efficacy

This study found that group polarization mediates the relationship between the spiral of silence and network cluster behavior. This indicates that, when participating in online cluster behavior, college students often observe, identify, and assess the prevailing attitudes and opinions of other Internet users, termed as the “opinion climate,” when discussing trending and sensitive topics or participating in negative online opinion events. If they perceive their views to be aligned with the majority or are widely accepted by the group, they are more likely to engage actively and boldly in discussions. Conversely, if their views diverge from those of the majority, they may opt to remain silent or conform to avoid social isolation. This results in a scenario where dominant viewpoints within the online group become more pronounced, while opposing viewpoints are marginalized and weakened, resulting in a downward spiral of opinion development (Sohn & Geidner, 2016). Ultimately, during group discussions and decision-making processes, certain opinions become amplified through interactive dialog among group members. Supportive views become more entrenched, while opposing views become more resistant, resulting in the phenomenon of group polarization (Sunstein, 2002). According to the Social Identity Theory (SIT), group polarization is a direct manifestation of the deepening of social identity, not only the convergence of group views, but also the radicalization of identity and the strengthening of positions. Group polarization causes the majority of college students’ views to become more dominant, while minority views are weakened (Dai et al., 2022). Additionally, the fragmentation of responsibility in online groups can result in risk transfer, whereby college students, motivated by specific objectives and interest demands, express and consolidate their opinions on a large scale in cyberspace. This can create online public opinion that influences or steers the development and resolution of events in their desired direction and may even lead to participation in excessive collective actions (Feng & Huang, 2021).

Social Identity Theory (SIT) holds that group efficacy is an individual’s belief in their ability to achieve goals (solve problems) through collective action. Only when individuals firmly believe that their group has the ability to change the status quo will a strong social identity be transformed into actual collective action. Further studies found that the interaction between group efficacy and group polarization significantly and negatively affected network cluster behavior. Group efficacy thus functions as a moderating variable in the relationship between group polarization and network cluster behavior, meaning that the latter part of the mediation path—spiral of silence → group polarization → network cluster behavior—is moderated by group efficacy. Specifically, when group efficacy is low, group polarization has a significant positive predictive effect on network clustering behavior. Conversely, when group efficacy is high, although group polarization still positively predicts network clustering behavior, the effect is less pronounced compared to the effect observed at low levels of group efficacy. This indicates that the spiral of silence occurring during college students’ discussions of trending and sensitive online topics or negative online opinion events leads to network clustering behavior, and varying levels of group efficacy modulate this process (van Zomeren et al., 2008).

Specifically, the effect is more pronounced in groups with low group efficacy compared to those with high group efficacy. This discrepancy may arise because groups of college students with low group efficacy are more likely to perceive their collective ability to express interests and demands, engage in joint solidarity, and influence or resolve events through online discussions and monitoring as limited (Meyer et al., 2022). Consequently, when group polarization occurs in these groups, it is more likely to result in extreme opinions, responsibility dispersion, risk transfer, and even the emergence of real-world group actions (Katz & Lazarsfeld, 1955).

## 6. Limitations and Future Directions

This study analyzed the group psychological characteristics and social psychological mechanisms of college students’ participation in online cluster behavior, which can provide theoretical and practical references for the government to strengthen cyberspace governance, universities to respond to and handle online cluster behavior, and to strengthen education and guidance for college students. However, this study still has the following deficiencies and limitations: First, we used cross-sectional questionnaire data, and the explanation of causality is weak. Future studies can consider using longitudinal research to explore group psychological characteristics and the social–psychological mechanism of network cluster behavior. Second, the representativeness of the sample for investigating network cluster behavior is not yet widespread enough. The network cluster behavior that occurs in the online social space involves complex participants and groups. College students are not the only group participating in network cluster behavior, but so do other social groups, involving different age groups, cultural knowledge, professions, and social backgrounds. However, this study focuses on students as the subject group, and due to the specificity of the sample, the research results obtained may affect the objectivity and universality of the conclusions when applied to other online social groups. College students may have significant differences in cognitive level, cultural literacy, and value judgments compared to other general social populations. Therefore, there may also be commonalities and differences in research conclusions, which need to be further improved in future research. At the same time, the number of lower-grade students in the sampling survey of this study is too large, which has the problem of over-representation, while the proportion of some graduates participating in the questionnaire survey due to academic pressure, graduation defense, and other factors is relatively small. Third, this study is mainly conducted through a questionnaire survey to obtain sample data for empirical analysis and research. In future research, we will analyze and verify the theoretical results combined with some typical cases of network cluster behavior or recent public opinion incidents. Fourth, in this study, emotional contagion and other group psychology are also influenced by traditional customs of different ethnic groups and cultural differences in different regions. However, the questionnaire’s design and quantitative analysis did not account for the factors of “cultural differences” or the applicability to other populations. Moreover, the subjects of this study are mainly Chinese college students; no national and cultural differences were investigated, and other non-group psychological factors—such as non-reaction bias, social expectation bias, socio-economic factors, and Internet usage habits—were not included in this study. These problems and shortcomings require further research and improvement.

## 7. Conclusions

This study analyzed the group psychological characteristics and social psychological mechanisms in network cluster behavior, and these research results have practical significance for universities to enhance the emergency response capabilities of college students in network cluster behavior.

Research has found that the action mobilization of opinion leaders plays a mediating role in the impact of emotional contagion on network cluster behavior. This suggests that college students are prone to emotional resonance and psychological identification when participating in discussions on sensitive topics or negative online public opinion events. At the same time, under the guidance and organizational mobilization of opinion leaders, it can affect the direction of public opinion development. (Liu et al., 2025) suggest that the government and universities can select and cultivate a team of opinion leaders with good political literacy and strong professional abilities from news media practitioners, experts and scholars, student backbones, etc. In dealing with sudden online public opinion situations and responding to online cluster behavior, the key factor of online opinion leaders can play a positive influence and guidance role.

Research has found that group polarization plays a mediating role between the spiral of silence and network clustering behavior. This suggests that in the process of the formation and fermentation of online public opinion, college students gather on a large scale around hot and sensitive topics in online public spaces, engage in joint discussions, express their interests and demands, and ultimately evolve into online cluster behavior, and even participate in real-life collective actions. But this process is not always the result of rational thinking among college students, but is influenced by the polarization psychology of the group. (Zhou, 2021) suggests that universities should actively intervene in response to online cluster behavior, release authoritative information, strengthen the ideological guidance and online public opinion guidance of college students, enhance rational thinking, judgment, and scientific cognitive analysis abilities, and reduce or avoid group polarization in the process of participating in group discussions.

Research has found that group efficacy moderates the relationship between group polarization and network cluster behavior. College students with a strong sense of group efficacy often believe that participating in discussions and expressing their interests through online group members can have a stronger belief and desire to influence or promote effective resolution of events (Shi & Cui, 2014). This suggests that universities can carry out targeted online ideological education and guidance based on different psychological and thinking characteristics of college students when dealing with and dealing with their online cluster behavior. It is recommended that universities provide classified education and guidance to college students with different performances in terms of group efficacy, self-evaluation, self-esteem, and other aspects. Education guides students to establish awareness of online laws, express their interests and demands reasonably, and prevent the occurrence of negative online cluster behavior.

## Figures and Tables

**Table 1:** The goodness of fit of the measurement model.

χ^2^/df	GFI	AGFI	IFI	CFI	NFI	RMSEA	RMR
3.665	0.976	0.965	0.973	0.973	0.963	0.035	0.025

**Table 2:** Descriptive statistics and correlations between variables.

Variables	M ± SD	1	2	3	4	5	6	7	8
1. Emotional infection	2.82 ± 0.75	1							
2. Depersonalization	2.63 ± 0.77	0.369 **	1						
3. Relative deprivation	2.18 ± 0.73	0.166 **	0.342 **	1					
4. Group polarization	2.39 ± 0.74	0.295 **	0.465 **	0.497 **	1				
5. Spiral of silence	2.56 ± 0.62	0.312 **	0.311 **	0.327 **	0.473 **	1			
6. Action mobilization	2.40 ± 0.73	0.205 **	0.376 **	0.341 **	0.474 **	0.390 **	1		
7. Network clustering behavior	2.57 ± 0.78	0.257 **	0.351 **	0.235 **	0.373 **	0.334 **	0.626 **	1	
8. Group efficacy	3.32 ± 0.65	0.142 **	0.102 **	−0.064 **	−0.01	0.074 **	0.101 **	0.188 **	1

N = 2391; ** *p* < 0.01.

**Table 3:** Testing the pathways of the mediation model.

	Model 1		Model 2		Model 3	
	*b(SE)*	*p*	*b(SE)*	*p*	*b*	*p*
Relative deprivation	0.078(0.023)	0.001			−0.026(0.020)	0.192
Depersonalization	0.229(0.023)	0.000			0.099(0.020)	0.000
Spiral of silence	0.176(0.018)	0.000			0.057(0.016)	0.000
Emotional infection	0.100(0.022)	0.000	0.301(0.031)	0.000	0.094(0.019)	0.000
Action mobilization					0.392(0.014)	0.000
R^2^	0.191		0.042		0.420	
F	125.801 ***		93.756 ***		308.330 ***	

Notes: *** *p* < 0.001.

**Table 4:** Testing the pathways of the mediation model with moderation.

	Model 1		Model 2		Model 3		Model 4	
	*b(SE)*	*p*	*b(SE)*	*p*	*b(SE)*	*p*	*b(SE)*	*p*
Relative deprivation	0.098(0.023)	0.000			0.054(0.024)	0.022	0.049(0.024)	0.038
Depersonalization	0.216(0.022)	0.000			0.180(0.023)	0.000	0.180(0.023)	0.000
Emotional infection	0.082(0.022)	0.000			0.070(0.022)	0.002	0.071(0.022)	0.001
Spiral of silence	0.169(0.018)	0.000	0.325(0.016)	0.000	0.142(0.018)	0.000	0.139(0.018)	0.000
Group efficacy	0.087(0.012)	0.000	0.028(0.011)	0.013	0.083(0.012)	0.000	0.167(0.033)	0.000
Group polarization					0.156(0.025)	0.000	0.408(0.094)	0.000
Group efficacy × group polarization							0.019(0.007)	0.006
R^2^	0.211		0.225		0.233		0.235	
F	114.193 ***		310.555 ***		107.568 ***		93.432 ***	

Notes: *** *p* < 0.001.

**Table 5:** Moderating effects at different levels of group efficacy.

	Group Efficacy Level	*β*	Se	t	*p*	LLCI	ULCI
Group polarization→network clustering behavior	−2.6002	0.4371	0.0275	15.8780	0.000	0.3831	0.4910
	0	0.3909	0.0205	19.1005	0.000	0.3508	0.4311
	2.6002	0.3448	0.0274	12.5668	0.000	0.2910	0.3986

## Data Availability

Data can be available on reasonable request from the authors.
